# 1-(2-Chloro­benzo­yl)-3-(pyrimidin-2-yl)thio­urea

**DOI:** 10.1107/S1600536812050118

**Published:** 2012-12-15

**Authors:** M. Khawar Rauf, Samad Yaseen, Masahiro Ebihara, Amin Badshah

**Affiliations:** a Department of Chemistry, Quaid-i-Azam University, Islamabad 45320, Pakistan; b Department of Chemistry, Faculty of Engineering, Gifu University Yanagido, Gifu 501-1193, Japan

## Abstract

In the title compound, C_12_H_9_ClN_4_OS, the carbonyl group is at a *cis* position with respect to the thio­urea unit. The dihedral angle between the phenyl and pyrimidine ring is 16.49 (6)°. An intra­molecular N—H⋯N hydrogen bond stabilizes the mol­ec­ular conformation. In the crystal, N—H⋯N, C—H⋯O and C—H⋯S hydrogen bonds generate chains along the *bc* axis.

## Related literature

For background to our work on structural and coordination chemistry of *N*,*N*′-disubstituted thio­ureas, see: Rauf *et al.* (2012 ▶). For a related structure, see: Sultana *et al.* (2007 ▶).
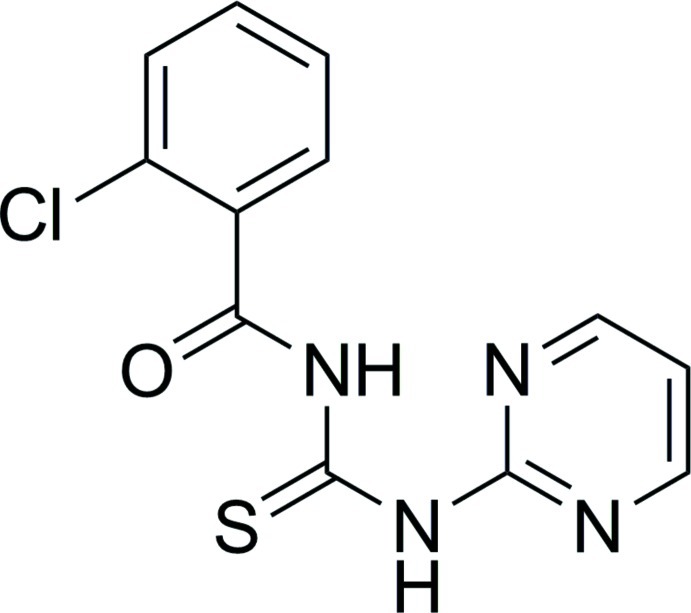


## Experimental

### 

#### Crystal data

C_12_H_9_ClN_4_OS
*M* *_r_* = 292.74
Triclinic, 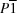

*a* = 7.167 (3) Å
*b* = 8.000 (4) Å
*c* = 11.252 (5) Å
α = 81.625 (14)°
β = 74.580 (12)°
γ = 83.979 (15)°
*V* = 613.8 (5) Å^3^
*Z* = 2
Mo *K*α radiation
μ = 0.48 mm^−1^
*T* = 123 K
0.30 × 0.26 × 0.18 mm

#### Data collection

Rigaku/MSC Mercury CCD diffractometer
4888 measured reflections
2759 independent reflections
2590 reflections with *I* > 2σ(*I*)
*R*_int_ = 0.032

#### Refinement

*R*[*F* ^2^ > 2σ(*F* ^2^)] = 0.034
*wR*(*F* ^2^) = 0.082
*S* = 1.07
2759 reflections
172 parameters
H-atom parameters constrained
Δρ_max_ = 0.29 e Å^−3^
Δρ_min_ = −0.34 e Å^−3^

### 

Data collection: *CrystalClear* (Molecular Structure Corporation & Rigaku, 2001 ▶); cell refinement: *CrystalClear* (Molecular Structure Corporation & Rigaku, 2001 ▶); data reduction: *CrystalClear*; program(s) used to solve structure: *SIR97* (Altomare *et al.*, 1999 ▶); program(s) used to refine structure: *SHELXL97* (Sheldrick, 2008 ▶); molecular graphics: *ORTEPII* (Johnson, 1976 ▶); software used to prepare material for publication: *Yadokari-XG 2009* (Kabuto *et al.*, 2009 ▶).

## Supplementary Material

Crystal structure: contains datablock(s) I, global. DOI: 10.1107/S1600536812050118/pv2612sup1.cif

Structure factors: contains datablock(s) I. DOI: 10.1107/S1600536812050118/pv2612Isup2.hkl

Supplementary material file. DOI: 10.1107/S1600536812050118/pv2612Isup3.cml

Additional supplementary materials:  crystallographic information; 3D view; checkCIF report

## Figures and Tables

**Table 1:** Hydrogen-bond geometry (Å, °)

*D*—H⋯*A*	*D*—H	H⋯*A*	*D*⋯*A*	*D*—H⋯*A*
N1—H1⋯N3	0.88	1.93	2.611 (2)	133
N2—H2⋯N4^i^	0.88	2.21	3.068 (2)	166
C11—H11⋯O1^ii^	0.95	2.28	3.200 (2)	163
C12—H12⋯S1^i^	0.95	2.77	3.568 (2)	142

Symmetry codes: (i) 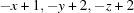; (ii) 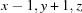.

**Fig. 1.**
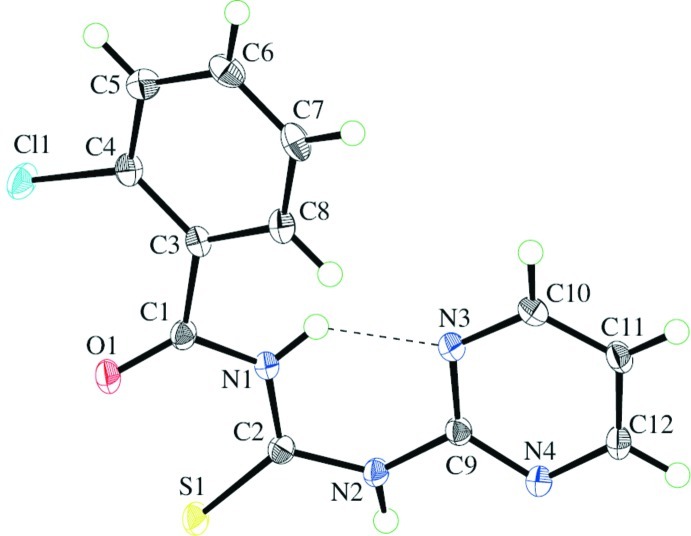
The molecular structure of the title compound with the atom numbering scheme. Displacement ellipsoids are drawn at the 50% probability level. H atoms are presented as small spheres of arbitrary radius. Hydrogen bond is shown as a dashed line.

**Fig. 2.**
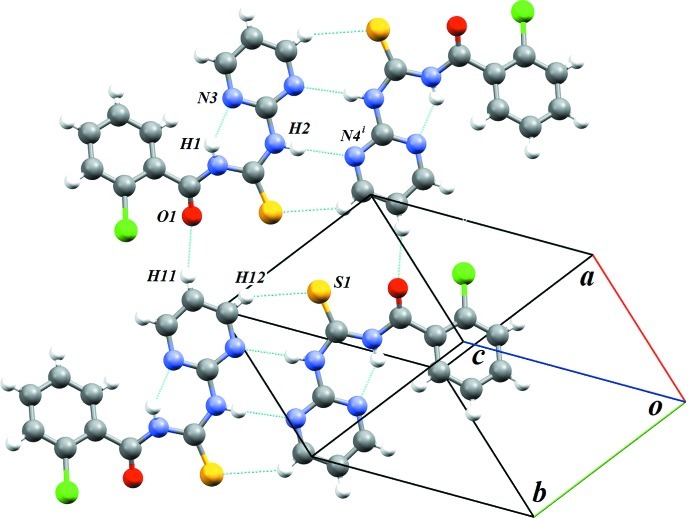
A view of the N—-H···N, C—H···O and C—H···S hydrogen bonds (dotted lines) in the crystal structure of the title compound.

## supplementary crystallographic information

### Comment

In continuation of our work on the structural and coordination chemistry of *N*,*N*'-disubstituted thioureas (Rauf *et al.*, 2012) the structure of the title compound (Fig. 1) is described in this article. The bond lengths and angles in the title compound agree very well with the corresponding bond legths and angles reported in a closely related compound (Sultana *et al.*, 2007). The molecule exists in its thione form with typical thiourea C—S and C—O bond distances, as well as shortened C—N bonds. The plane containing the S1, C2, N1 & N2 atoms is almost parallel to the pyrimidine ring, forming a dihedral angle of 9.09 (13)°. The molecules also feature intra & intermolecular N—H···N, C—H···O and C—H···S hydrogen bonds (Table 1 & Fig. 2).

### Experimental

Freshly prepared 2-chlorobenzoylisothiocyanate (1.98 g, 10 mmol) was dissolved in tetrahydrofuran (35 ml) and stirred for 40 minutes. Afterwards neat 2-aminopyrimidine (1.0 g, 10 mmol) was added and the resulting mixture was stirred for 1 h. The reaction mixture was then poured into acidified water and stirred well. The solid product was separated and washed with deionized water and purified by recrystallization from chloroform to give fine crystals of the title compound, with an overall yield of 92% (2.8 g).

### Refinement

All H atoms were positioned geometrically and refined using a riding model, with C—H = 0.95 and N—H = 0.88 Å, and *U*_iso_(H) set at 1.2*U*_eq_(C/N).

### Crystal data

**Table:**

C_12_H_9_ClN_4_OS	*Z* = 2
*M**_r_* = 292.74	*F*(000) = 300
Triclinic, *P*1	*D*_x_ = 1.584 Mg m^−^^3^
Hall symbol: -P 1	Mo *K*α radiation, λ = 0.71070 Å
*a* = 7.167 (3) Å	Cell parameters from 2102 reflections
*b* = 8.000 (4) Å	θ = 3.0–27.5°
*c* = 11.252 (5) Å	µ = 0.48 mm^−^^1^
α = 81.625 (14)°	*T* = 123 K
β = 74.580 (12)°	Block, yellow
γ = 83.979 (15)°	0.30 × 0.26 × 0.18 mm
*V* = 613.8 (5) Å^3^	

### Data collection

**Table:**

Rigaku/MSC Mercury CCD diffractometer	2590 reflections with *I* > 2σ(*I*)
Radiation source: Rotating Anode	*R*_int_ = 0.032
Graphite Monochromator monochromator	θ_max_ = 27.5°, θ_min_ = 3.0°
Detector resolution: 14.62 pixels mm^-1^	*h* = −8→9
ω scans	*k* = −9→10
4888 measured reflections	*l* = −14→14
2759 independent reflections	

### Refinement

**Table:**

Refinement on *F*^2^	Primary atom site location: structure-invariant direct methods
Least-squares matrix: full	Secondary atom site location: difference Fourier map
*R*[*F*^2^ > 2σ(*F*^2^)] = 0.034	Hydrogen site location: inferred from neighbouring sites
*wR*(*F*^2^) = 0.082	H-atom parameters constrained
*S* = 1.07	*w* = 1/[σ^2^(*F*_o_^2^) + (0.0275*P*)^2^ + 0.4951*P*] where *P* = (*F*_o_^2^ + 2*F*_c_^2^)/3
2759 reflections	(Δ/σ)_max_ = 0.001
172 parameters	Δρ_max_ = 0.29 e Å^−^^3^
0 restraints	Δρ_min_ = −0.34 e Å^−^^3^

### Special details

**Table:**

Geometry. All e.s.d.'s (except the e.s.d. in the dihedral angle between two l.s. planes) are estimated using the full covariance matrix. The cell e.s.d.'s are taken into account individually in the estimation of e.s.d.'s in distances, angles and torsion angles; correlations between e.s.d.'s in cell parameters are only used when they are defined by crystal symmetry. An approximate (isotropic) treatment of cell e.s.d.'s is used for estimating e.s.d.'s involving l.s. planes.
Refinement. Refinement of *F*^2^ against ALL reflections. The weighted *R*-factor *wR* and goodness of fit *S* are based on *F*^2^, conventional *R*-factors *R* are based on *F*, with *F* set to zero for negative *F*^2^. The threshold expression of *F*^2^ > σ(*F*^2^) is used only for calculating *R*-factors(gt) *etc*. and is not relevant to the choice of reflections for refinement. *R*-factors based on *F*^2^ are statistically about twice as large as those based on *F*, and *R*- factors based on ALL data will be even larger.

### Fractional atomic coordinates and isotropic or equivalent isotropic displacement parameters (Å^2^)

**Table:**

	*x*	*y*	*z*	*U*_iso_*/*U*_eq_	
C1	0.5699 (2)	0.40086 (19)	0.77222 (14)	0.0168 (3)	
O1	0.66497 (17)	0.29064 (14)	0.81927 (11)	0.0232 (3)	
N1	0.54980 (19)	0.57016 (16)	0.79213 (12)	0.0174 (3)	
H1	0.4808	0.6385	0.7489	0.021*	
C2	0.6242 (2)	0.64410 (19)	0.87083 (14)	0.0167 (3)	
S1	0.80674 (6)	0.56064 (5)	0.93013 (4)	0.02194 (12)	
N2	0.53983 (18)	0.80178 (16)	0.89581 (12)	0.0172 (3)	
H2	0.5987	0.8564	0.9362	0.021*	
C3	0.4553 (2)	0.36848 (18)	0.68461 (14)	0.0160 (3)	
C4	0.5267 (2)	0.25791 (19)	0.59525 (15)	0.0175 (3)	
C5	0.4187 (2)	0.2281 (2)	0.51587 (15)	0.0206 (3)	
H5	0.4713	0.1549	0.4540	0.025*	
C6	0.2331 (2)	0.3058 (2)	0.52718 (16)	0.0225 (3)	
H6	0.1586	0.2857	0.4730	0.027*	
C7	0.1569 (2)	0.4122 (2)	0.61713 (16)	0.0222 (3)	
H7	0.0290	0.4634	0.6260	0.027*	
C8	0.2671 (2)	0.44417 (19)	0.69453 (15)	0.0190 (3)	
H8	0.2141	0.5187	0.7554	0.023*	
Cl1	0.75833 (5)	0.15669 (5)	0.57492 (4)	0.02521 (12)	
C9	0.3753 (2)	0.88839 (19)	0.86695 (14)	0.0162 (3)	
N3	0.2989 (2)	0.83057 (17)	0.78621 (13)	0.0208 (3)	
C10	0.1374 (2)	0.9160 (2)	0.76400 (15)	0.0215 (3)	
H10	0.0770	0.8766	0.7087	0.026*	
C11	0.0568 (2)	1.0582 (2)	0.81851 (15)	0.0191 (3)	
H11	−0.0576	1.1179	0.8028	0.023*	
C12	0.1518 (2)	1.10993 (19)	0.89792 (15)	0.0185 (3)	
H12	0.1015	1.2096	0.9356	0.022*	
N4	0.31077 (18)	1.02648 (16)	0.92412 (12)	0.0169 (3)	

### Atomic displacement parameters (Å^2^)

**Table:**

	*U*^11^	*U*^22^	*U*^33^	*U*^12^	*U*^13^	*U*^23^
C1	0.0139 (7)	0.0176 (7)	0.0177 (7)	0.0017 (5)	−0.0021 (5)	−0.0037 (6)
O1	0.0235 (6)	0.0197 (5)	0.0287 (6)	0.0079 (4)	−0.0125 (5)	−0.0064 (5)
N1	0.0190 (6)	0.0162 (6)	0.0180 (6)	0.0043 (5)	−0.0074 (5)	−0.0039 (5)
C2	0.0149 (7)	0.0182 (7)	0.0160 (7)	0.0011 (5)	−0.0025 (5)	−0.0029 (5)
S1	0.01718 (19)	0.0234 (2)	0.0285 (2)	0.00729 (14)	−0.01126 (16)	−0.01008 (16)
N2	0.0159 (6)	0.0175 (6)	0.0198 (6)	0.0030 (5)	−0.0070 (5)	−0.0060 (5)
C3	0.0147 (7)	0.0139 (6)	0.0184 (7)	0.0000 (5)	−0.0043 (6)	0.0001 (5)
C4	0.0136 (7)	0.0165 (7)	0.0210 (7)	−0.0011 (5)	−0.0028 (6)	−0.0009 (6)
C5	0.0217 (8)	0.0207 (7)	0.0197 (7)	−0.0044 (6)	−0.0048 (6)	−0.0024 (6)
C6	0.0210 (8)	0.0240 (8)	0.0248 (8)	−0.0064 (6)	−0.0112 (6)	0.0029 (6)
C7	0.0154 (7)	0.0210 (7)	0.0298 (8)	−0.0003 (6)	−0.0085 (6)	0.0029 (6)
C8	0.0156 (7)	0.0166 (7)	0.0235 (8)	0.0009 (6)	−0.0041 (6)	−0.0010 (6)
Cl1	0.01698 (19)	0.0298 (2)	0.0311 (2)	0.00670 (15)	−0.00699 (15)	−0.01528 (17)
C9	0.0153 (7)	0.0164 (7)	0.0156 (7)	0.0008 (5)	−0.0029 (5)	−0.0011 (5)
N3	0.0226 (7)	0.0209 (6)	0.0213 (7)	0.0062 (5)	−0.0107 (5)	−0.0063 (5)
C10	0.0228 (8)	0.0230 (8)	0.0210 (8)	0.0032 (6)	−0.0113 (6)	−0.0035 (6)
C11	0.0160 (7)	0.0197 (7)	0.0202 (7)	0.0033 (6)	−0.0053 (6)	0.0001 (6)
C12	0.0165 (7)	0.0151 (7)	0.0212 (7)	0.0023 (5)	−0.0021 (6)	−0.0010 (6)
N4	0.0155 (6)	0.0157 (6)	0.0189 (6)	0.0005 (5)	−0.0036 (5)	−0.0026 (5)

### Geometric parameters (Å, º)

**Table:**

C1—O1	1.2051 (19)	C6—C7	1.380 (2)
C1—N1	1.391 (2)	C6—H6	0.9500
C1—C3	1.504 (2)	C7—C8	1.386 (2)
N1—C2	1.374 (2)	C7—H7	0.9500
N1—H1	0.8800	C8—H8	0.9500
C2—N2	1.374 (2)	C9—N3	1.334 (2)
C2—S1	1.6596 (16)	C9—N4	1.338 (2)
N2—C9	1.3937 (19)	N3—C10	1.344 (2)
N2—H2	0.8800	C10—C11	1.371 (2)
C3—C4	1.395 (2)	C10—H10	0.9500
C3—C8	1.403 (2)	C11—C12	1.386 (2)
C4—C5	1.386 (2)	C11—H11	0.9500
C4—Cl1	1.7420 (17)	C12—N4	1.339 (2)
C5—C6	1.390 (2)	C12—H12	0.9500
C5—H5	0.9500		
			
O1—C1—N1	125.24 (14)	C7—C6—H6	120.0
O1—C1—C3	122.86 (14)	C5—C6—H6	120.0
N1—C1—C3	111.90 (12)	C6—C7—C8	119.97 (15)
C2—N1—C1	128.18 (13)	C6—C7—H7	120.0
C2—N1—H1	115.9	C8—C7—H7	120.0
C1—N1—H1	115.9	C7—C8—C3	121.20 (15)
N1—C2—N2	114.75 (13)	C7—C8—H8	119.4
N1—C2—S1	125.09 (12)	C3—C8—H8	119.4
N2—C2—S1	120.12 (12)	N3—C9—N4	126.22 (14)
C2—N2—C9	129.89 (13)	N3—C9—N2	118.96 (14)
C2—N2—H2	115.1	N4—C9—N2	114.82 (13)
C9—N2—H2	115.1	C9—N3—C10	116.63 (14)
C4—C3—C8	117.63 (14)	N3—C10—C11	122.19 (15)
C4—C3—C1	122.04 (13)	N3—C10—H10	118.9
C8—C3—C1	120.26 (14)	C11—C10—H10	118.9
C5—C4—C3	121.41 (14)	C10—C11—C12	116.25 (14)
C5—C4—Cl1	117.02 (12)	C10—C11—H11	121.9
C3—C4—Cl1	121.54 (12)	C12—C11—H11	121.9
C4—C5—C6	119.70 (15)	N4—C12—C11	123.30 (14)
C4—C5—H5	120.2	N4—C12—H12	118.3
C6—C5—H5	120.2	C11—C12—H12	118.3
C7—C6—C5	120.06 (15)	C9—N4—C12	115.35 (13)
			
O1—C1—N1—C2	−3.0 (3)	C4—C5—C6—C7	0.0 (2)
C3—C1—N1—C2	176.24 (14)	C5—C6—C7—C8	1.3 (2)
C1—N1—C2—N2	−163.48 (14)	C6—C7—C8—C3	−0.9 (2)
C1—N1—C2—S1	18.9 (2)	C4—C3—C8—C7	−0.9 (2)
N1—C2—N2—C9	9.3 (2)	C1—C3—C8—C7	−178.10 (14)
S1—C2—N2—C9	−172.89 (13)	C2—N2—C9—N3	−12.7 (2)
O1—C1—C3—C4	−39.8 (2)	C2—N2—C9—N4	168.18 (15)
N1—C1—C3—C4	140.94 (14)	N4—C9—N3—C10	−2.7 (2)
O1—C1—C3—C8	137.32 (16)	N2—C9—N3—C10	178.32 (14)
N1—C1—C3—C8	−41.99 (19)	C9—N3—C10—C11	1.7 (2)
C8—C3—C4—C5	2.2 (2)	N3—C10—C11—C12	0.2 (2)
C1—C3—C4—C5	179.40 (14)	C10—C11—C12—N4	−1.5 (2)
C8—C3—C4—Cl1	−179.94 (11)	N3—C9—N4—C12	1.5 (2)
C1—C3—C4—Cl1	−2.8 (2)	N2—C9—N4—C12	−179.46 (13)
C3—C4—C5—C6	−1.8 (2)	C11—C12—N4—C9	0.7 (2)
Cl1—C4—C5—C6	−179.74 (12)		

### Hydrogen-bond geometry (Å, º)

**Table:**

*D*—H···*A*	*D*—H	H···*A*	*D*···*A*	*D*—H···*A*
N1—H1···N3	0.88	1.93	2.611 (2)	133
N2—H2···N4^i^	0.88	2.21	3.068 (2)	166
C11—H11···O1^ii^	0.95	2.28	3.200 (2)	163
C12—H12···S1^i^	0.95	2.77	3.568 (2)	142

Symmetry codes: (i) −*x*+1, −*y*+2, −*z*+2; (ii) *x*−1, *y*+1, *z*.
